# One new species of *Fannia* (Diptera, Fanniidae) from Yunnan, China with a key to the *Fanniafuscinata*-group in China

**DOI:** 10.3897/BDJ.9.e72444

**Published:** 2021-11-22

**Authors:** Shuyu Wei, Mingfu Wang, Dong Zhang

**Affiliations:** 1 School of Ecology and Nature Conservation, Beijing Forestry University, Beijing, China School of Ecology and Nature Conservation, Beijing Forestry University Beijing China; 2 Institute of Entomology, Shenyang Normal University, Shenyang, China Institute of Entomology, Shenyang Normal University Shenyang China

**Keywords:** new species, *Fanniafuscinata*-group, Yunnan, male terminalia

## Abstract

**Background:**

The Fannidae includes over 400 described species, mainly known from the Holarctic Region. The number of species in the Oriental Region are underestimated. The *Fanniafuscinata*-group was established by Wang et al. in 2011, consisting of nine species at present.

**New information:**

A new species of the genus *Fannia* (Diptera, Fanniidae) is described from Yunnan, part of the Oriental Region in China, namely *Fanniamenglaensis* **sp. nov.** The detailed description, photographs and drawings of adults and male terminalia of *F.menglaensis* **sp. nov.** are provided. All specimens are preserved in the Museum of Beijing Forestry University.

## Introduction

The Fanniidae is a group of Calyptratae and the sister group of a large clade including Muscidae and Anthomyiidae and the Oestroidea (Kutty et al. 2019). More than 400 species of this family have been recorded all over the world, belonging to five genera. The fanniids are mostly found in forested areas and the males are often seen in clearings inside the forests.

The *Fanniafuscinata*-group was separated from the *metallipennis*-group and erected as an independent group by Wang et al. (2011) because of the characteristic and complex branched processes of cercus and surstylus. As three of twelve species, *Fanniahuaxiia* Feng and Xue 2000a, *F. quinquirmula Feng 2002* and *F. xui Feng 2003* would be regarded as synonymies (unpublished), only nine species occur in the world (Wang et al. 2011). Apart from *F.pileatus*, other species were found in the Palaearctic Region. Before the present contribution, eight species of the *F.fuscinata*-group were known in China (Wang et al. 2011). However, the number of species in Oriental China is underestimated. Yunnan, in the southwest of China, is one of the greatest biodiversity hotspots in the world (Myers et al. 2000) and is part of the Oriental Region in China, which has many species yet to be described. We found a new species in this region and we are describing it herein, providing an identification key to species of the *Fanniafuscinata*-group in China.

## Materials and methods

Terminology follows Cumming and Wood (2017) and Stuckenberg (1999). A series of photographs of continuous sequences were taken by a Canon 750D digital camera, coupled with ZEISS SteREO Discovery. Prior to illustration, the abdomens were detached from pinned male specimens, placed in 10% near-boiling potassium hydroxide (KOH) for about 5 min, rinsed in distilled water, then placed in 10% acetic acid to stabilise and partly dissected before rinsing in distilled water. The terminalia were transferred to glycerol, where final dissections were performed. V20 and stacked Helicon Focus 3.20 Free for Windows were used to combine images with more field depth. The digital images were labelled on Windows 10 platform by Adobe Photoshop CC 2018 for Windows. All type specimens of this new species, examined in this paper, including the types of the new species, are deposited in the Museum of Beijing Forestry University, Beijing, China (MBFU).

## Taxon treatments

### Fannia menglaensis sp. n.

F55DBEB0-6ABC-5DE0-ABDB-9324B6AC18AE

4DB6D6FF-B2D9-45DB-A53B-ACEBFDF3233E

#### Materials

- **Type status:** Holotype. **Occurrence:** recordedBy: D. Zhang & J. R. Zhang; individualID: the Museum of Beijing Forestry University, Beijing, China; sex: male; lifeStage: adult; **Location:** country: China; countryCode: CN; stateProvince: Yunnan; county: Xishuangbanna; municipality: Mengla; locality: Wild Elephant Valley; verbatimLatitude: 22°10'22.68"N; verbatimLongitude: 100°51'29.39"E; **Event:** year: 2018; month: 2; day: 13; **Record Level:** basisOfRecord: PreservedSpecimen
- **Type status:** Paratype. **Occurrence:** recordedBy: D. Zhang & J. R. Zhang; individualID: the Museum of Beijing Forestry University, Beijing, China; sex: female; lifeStage: adult; **Location:** country: China; countryCode: CN; stateProvince: Yunnan; county: Xishuangbanna; municipality: Mengla; locality: Wild Elephant Valley; verbatimLatitude: 22°10'22.68"N; verbatimLongitude: 100°51'29.39"E; **Event:** year: 2018; month: 2; day: 13; **Record Level:** basisOfRecord: PreservedSpecimen
- **Type status:** Paratype. **Occurrence:** recordedBy: D. Zhang & J. R. Zhang; individualID: the Museum of Beijing Forestry University, Beijing, China; sex: male; lifeStage: adult; **Location:** country: China; countryCode: CN; stateProvince: Yunnan; county: Xishuangbanna; municipality: Mengla; locality: Wild Elephant Valley; verbatimLatitude: 22°10'22.68"N; verbatimLongitude: 100°51'29.39"E; **Event:** year: 2018; month: 2; day: 13; **Record Level:** basisOfRecord: PreservedSpecimen
- **Type status:** Paratype. **Occurrence:** recordedBy: D. Zhang & J. R. Zhang; individualID: the Museum of Beijing Forestry University, Beijing, China; sex: male; lifeStage: adult; **Location:** country: China; countryCode: CN; stateProvince: Yunnan; county: Xishuangbanna; municipality: Mengla; locality: Wild Elephant Valley; verbatimLatitude: 22°10'22.68"N; verbatimLongitude: 100°51'29.39"E; **Event:** year: 2018; month: 2; day: 13; **Record Level:** basisOfRecord: PreservedSpecimen
- **Type status:** Paratype. **Occurrence:** recordedBy: D. Zhang & J. R. Zhang; individualID: the Museum of Beijing Forestry University, Beijing, China; sex: male; lifeStage: adult; **Location:** country: China; countryCode: CN; stateProvince: Yunnan; county: Xishuangbanna; municipality: Mengla; locality: Wild Elephant Valley; verbatimLatitude: 22°10'22.68"N; verbatimLongitude: 100°51'29.39"E; **Event:** year: 2018; month: 2; day: 13; **Record Level:** basisOfRecord: PreservedSpecimen
- **Type status:** Paratype. **Occurrence:** recordedBy: D. Zhang & J. R. Zhang; individualID: the Museum of Beijing Forestry University, Beijing, China; sex: male; lifeStage: adult; **Location:** country: China; countryCode: CN; stateProvince: Yunnan; county: Xishuangbanna; municipality: Mengla; locality: Wild Elephant Valley; verbatimLatitude: 22°10'22.68"N; verbatimLongitude: 100°51'29.39"E; **Event:** year: 2018; month: 2; day: 13; **Record Level:** basisOfRecord: PreservedSpecimen
- **Type status:** Paratype. **Occurrence:** recordedBy: D. Zhang & J. R. Zhang; individualID: the Museum of Beijing Forestry University, Beijing, China; sex: male; lifeStage: adult; **Location:** country: China; countryCode: CN; stateProvince: Yunnan; county: Xishuangbanna; municipality: Mengla; locality: Wild Elephant Valley; verbatimLatitude: 22°10'22.68"N; verbatimLongitude: 100°51'29.39"E; **Event:** year: 2018; month: 2; day: 13; **Record Level:** basisOfRecord: PreservedSpecimen
- **Type status:** Paratype. **Occurrence:** recordedBy: D. Zhang & J. R. Zhang; individualID: the Museum of Beijing Forestry University, Beijing, China; sex: male; lifeStage: adult; **Location:** country: China; countryCode: CN; stateProvince: Yunnan; county: Xishuangbanna; municipality: Mengla; locality: Wild Elephant Valley; verbatimLatitude: 22°10'22.68"N; verbatimLongitude: 100°51'29.39"E; **Event:** year: 2018; month: 2; day: 13; **Record Level:** basisOfRecord: PreservedSpecimen

#### Description

**Figure 1.**
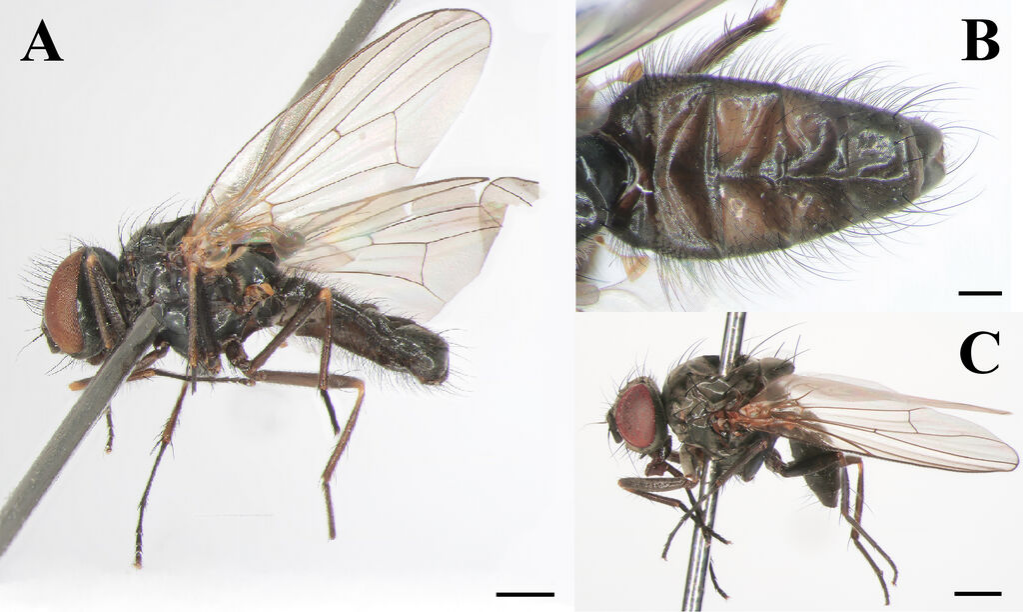
*Fanniamenglaensis* sp. nov. from Yunnan, China. **A** Male habitus, lateral view (BFU–10730, holotype); **B** Male abdomen, dorsal view (BFU–10730, holotype); **C** Female habitus, lateral view (BFU–10730, paratype). Scale bars: **A** = 1.00 mm; **B** = 0.50 mm; **C** = 1.00 mm.

Male: Body length 5.5–6.0 mm (Fig. 1A). Eye bare. Fronto-orbital plate and parafacial with dense greyish pollinosity; frons almost as wide as the distance between two posterior ocelli at the narrowest point. Frontal vitta black, the narrowest point as 1/2 the width of fronto-orbital plate. Frontal setae twenty, nearly reaching ocellar triangle. Postocular setae in one row, hair-like, slender and forward, occipital setae behind the postocular setae on vertex in one shorter row. Parafacial bare and black, at middle as 1/2 the width of fronto-orbital plate. Antenna black, postpedicel 2.5x longer than wide, arista pubescent, swollen in basal part, the longest individual hair shorter than basal aristal width. Epistoma not projecting; subvibrissal setae in one row, laterally with one row of short setae. Genae with black setae, greyish pollinosity. Palpus black, claviform, longer than the length of prementum.

Thorax translucent yellow black, notum without distinct vitta; preustural acrostichal seta in four rows, only prescutellar pairs slightly stout, dorsocentrals 2+3, intra-alars 0+2, supra-alars 2, scutellum with widely spread fine setae in the dorsal surface, except median parts; notopleuron without seta. Katepisternal setae 1:1. Anterior spiracle brown. Calypters yellow, the lower one slightly projecting beyond the upper one.

Wings light brown; veins dark brown, tegula brown; basicosta brown, costal spine inconspicuous. Crossveins without obvious cloud; haltere yellow.

Knees yellow, other parts of legs black. Fore femur with complete posteroventral seta and posterodorsal seta rows, fore tibia with one apical posterior seta; mid-coxa without any hooked spines or spine-like setae on lower and outer margins; mid-femur with complete anteroventral seta and posteroventral seta rows, becoming gradually denser towards apex (Fig. 3C), then with 8 thorn-like posterodorsal seta in apical part; mid-tibia with one anterodorsal seta, one subapical anteroventral seta and posteroventral seta (Fig. 3D); mid first tarsomere without basal tooth-like spines on ventral surface; hind femur with a pre-apical anteroventral seta, without posterior seta and posteroventral seta rows; hind tibia with one submedian anteroventral seta, one submedian anterodorsal seta and one subapical dorsal seta.

Abdomen black, long and flattened. Each tergite with long lateral marginal setae. Syntergite 1+2 to tergite 4, each with one median inverted black triangular vitta (Fig. 1B). Surstylus separated into two branches, anterior branch of surstylus with spiral and twist, with hook-like projection, then with some setae on turning point and apical, posterior branch of surstylus with hand-like projection (Fig. 4A); cercus separated into six branches, anterior two branches bacilliform, profoundly curved inwards, with swollen marginals, median two branches bacilliform, with length as 1/2 the length of posterior branches, posterior two branches long and slender, with hook-like projection on its lower margin curved outwards. (Fig. 4C).

**Figure 2.**
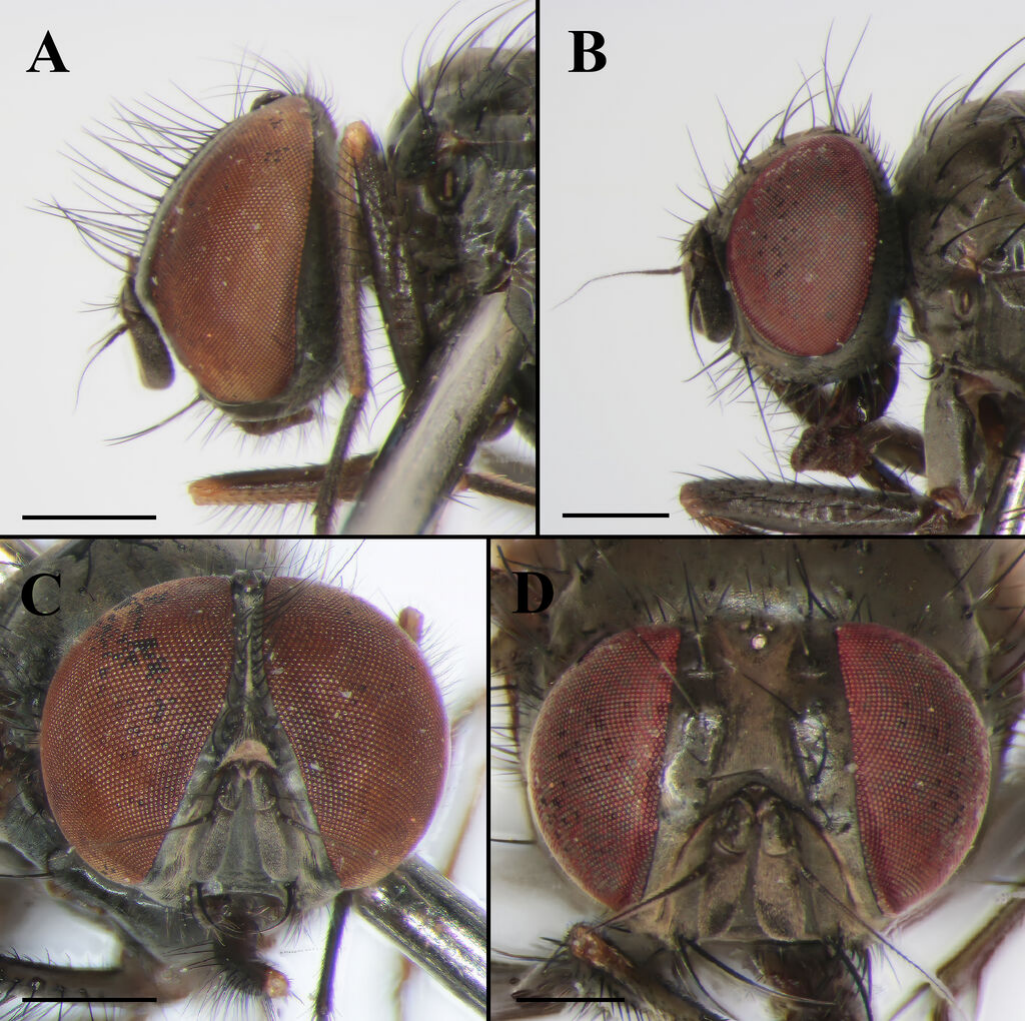
*Fanniamenglaensis* sp. nov. from Yunnan, China. **A** Male head, lateral view (BFU–10730, holotype); **B** Female head, lateral view (BFU–10731, paratype); **C** Male head, anterior view (holotype, BFU–10730); **D** Female head, anterior view (BFU–10731, paratype). Scale bars: **A**–**C** = 1.00 mm; **D** = 0.50 mm.

Female: Body length 5.2 mm (Fig. 1C). Eye bare. Fronto-orbital plate partly shining, with dense greyish pollinosity, parafacial with dense greyish pollinosity; frons at middle about 2/5 as wide as the distance between the width of eyes. Parafacial as 1/3 the width of fronto-orbital plate. Antenna black, postpedicel 2.0x longer than wide (Fig. 2D). Legs entirely black. Other morphological characteristics are similar to those of the male.

#### Etymology

The specific epithet refers to the name of the type locality: Mengla.

#### Distribution

The species are at present known only from Yunnan, China.

## Identification Keys

### Key to the known species of the *Fanniafuscinata*-group in China (male)

**Table:** 

1	Fore and mid-legs entirely yellow	2
–	Fore and mid-legs entirely black or partly black	3
2	Scutellum with four profoundly dark vertical stripes, surstylus in lateral view without branches	***Fanniapolystylata****Xue et al. 1997* Distribution: China: Shanxi .
–	Scutellum without dark vertical stripes, surstylus in lateral view with branches	***Fanniaflavifuscinnta***Xue 1997 Distribution: China: Hubei, Sichuan.
3	Mid-tibia with only one anterodorsal seta	4
–	Mid-tibia with two or more anterodorsal seta, at least on one side with two anterodorsal seta	6
4	Frontal setae twenty; postpedicel 2.50x longer than wide; eye bare; cercus with projecting process	5
–	Frontal setae twelve or thirteen; postpedicel 2.00x longer than wide; eye with hairs; anterior branch of cercus without projecting process	***Fanniamaximiguttatus***Feng and Xue 2006 Distribution: China: Sichuan.
5	Posterior branches of cercus with projection on its lower margin strongly curved inwards	***Fanniapileatus***Xue et al. 2001 Distribution: China: Yunnan.
–	Posterior branches of cercus with the projection on its lower margin curved outwards	***Fanniamenglaensis* sp. nov.**
6	Eye with ommatrichia	7
–	Eye bare	***Fanniascissifolia***Xue et al. 1997 Distribution: China: Hunan.
7	Hind femur with only one anteroventral seta; calypters brown; surstylus with sharp anterior branch	***Fanniapolystylodes***Feng and Xue 2000b Distribution: China: Sichuan.
–	Hind femur with two anteroventral seta; calypters from white to yellow; anterior branch of surstylus with incision or not	8
8	Presutural acr two rows; frontal setae thirteen or fourteen pairs; cercus with projecting process from its postsutural part	***Fanniatriaenocera***Xue and Yang 2000 Distribution: China: Zhejiang.
–	Presutural acrostichal seta three rows; frontal setae ten to twelve pairs; cercus with projecting process from its median part in ventral view	***Fanniafuscinata***Chillcott 1961 Disribution: China: Yunnan, Sichuan.

## Discussion

**Figure 3.**
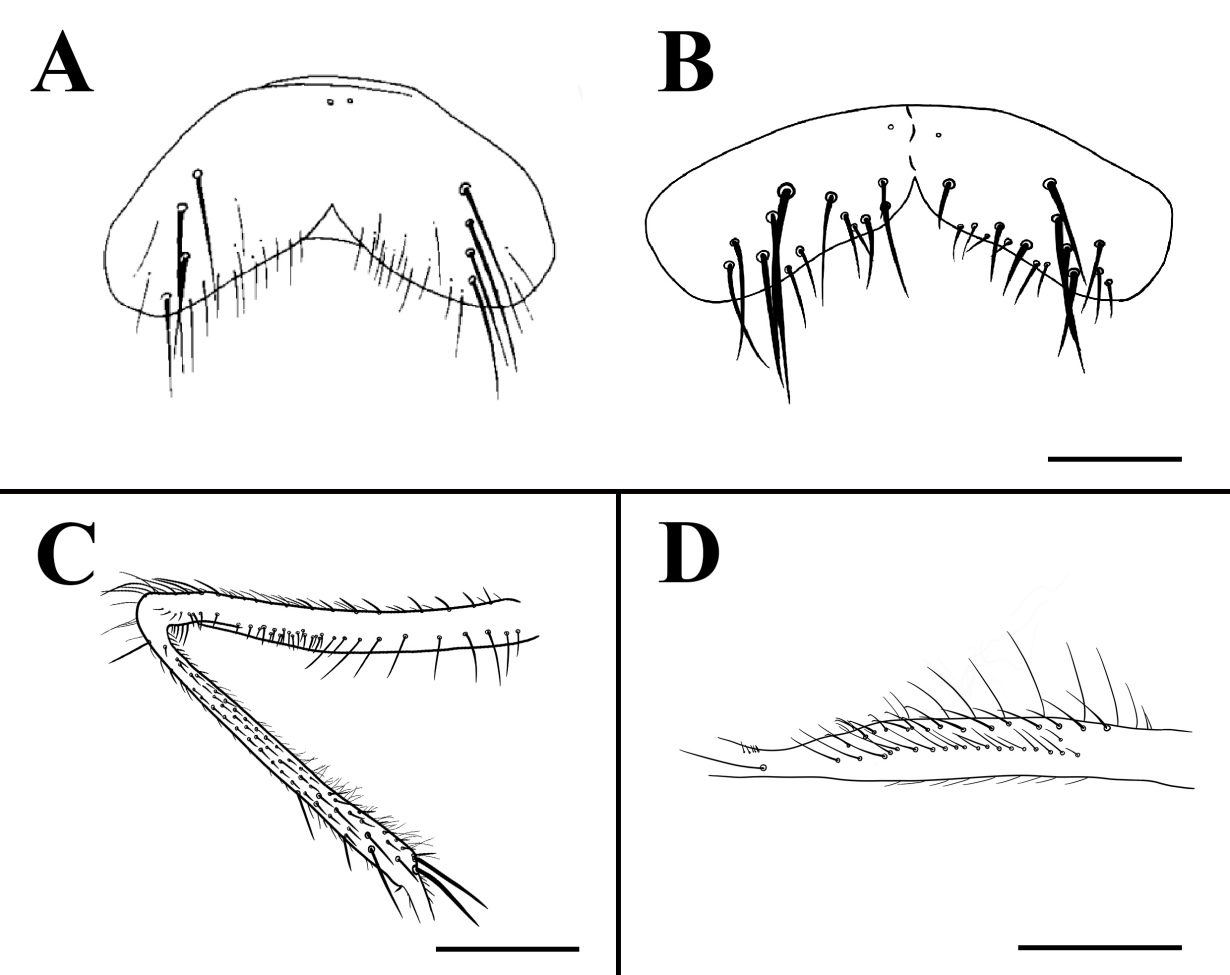
**A** Sternite 5 of *Fanniapileatus* Xue et al. 2001, male, ventral view; **B** Sternite 5 of *Fanniamenglaensis* sp. nov. from Yunnan, China, male, ventral view (BFU–10732, paratype); **C** Mid-femur and tibia of *Fanniamenglaensis* sp. nov. from Yunnan, China, male, anterior view (BFU–10730, holotype); **D** Mid-femur of *Fanniamenglaensis* sp. nov. from Yunnan, China, male, posteroventral view (BFU–10730, holotype). Scale bars: **B** = 0.30 mm; **C**, **D** = 0.50 mm.

**Figure 4.**
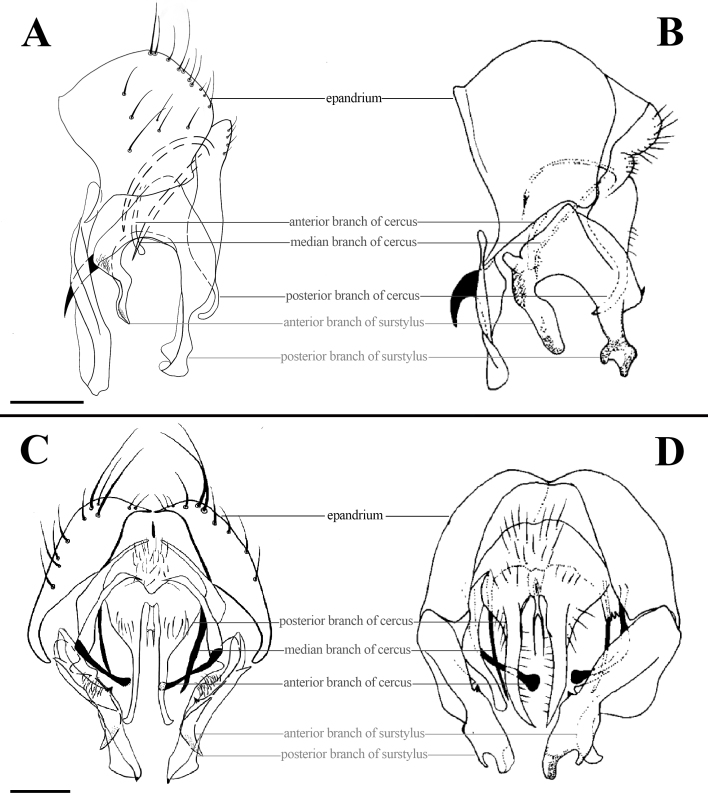
**A** Male terminalia of *Fanniamenglaensis* sp. nov. from Yunnan, China, lateral view (BFU–10732, paratype); **B** Male terminalia of *F.pileatus* Xue et al. 2001, lateral view; **C** Male terminalia of *Fanniamenglaensis* sp. nov. from Yunnan, China, ventral view (BFU–10732, paratype); **D** Male terminalia of *F.pileatus* Xue et al. 2001, ventral view. Scale bars: **A** = 0.30 mm; **C** = 0.30 mm.

*Fanniamenglaensis* sp. nov. is most similar to *F.pileatus* Xue et al. 2001, especially the shape of surstylus, but the new species can be distinguished by the shape of sternite 5, branches of surstylus and the shape of cercus. Sternite 5 of *F.menglaensis* sp. nov. is slender and flat, with the length 4.00x longer than middle width (Fig. 3B); the anterior branch of surstylus does not have a protruding process; and the length of posterior branch of surstylus is 4.50x longer than width (Fig. 4A); and the posterior two branches of cercus with the hook-like projection on its lower margin are strongly curved outwards (Fig. 4C).

## Supplementary Material

XML Treatment for Fannia menglaensis
